# BART-Seq: cost-effective massively parallelized targeted sequencing for genomics, transcriptomics, and single-cell analysis

**DOI:** 10.1186/s13059-019-1748-6

**Published:** 2019-08-06

**Authors:** Fatma Uzbas, Florian Opperer, Can Sönmezer, Dmitry Shaposhnikov, Steffen Sass, Christian Krendl, Philipp Angerer, Fabian J. Theis, Nikola S. Mueller, Micha Drukker

**Affiliations:** 1 Institute of Stem Cell Research, Helmholtz Center Munich, 85764 Neuherberg, Germany; 2 Genome Biology Unit, European Molecular Biology Laboratory, 69117 Heidelberg, Germany; 3 Institute of Computational Biology, Helmholtz Center Munich, 85764 Neuherberg, Germany; 4 Department of Mathematics, Technical University Munich, 85748 Garching, Germany

**Keywords:** Barcoding, Single-cell RNA sequencing, Targeted transcriptomics, High-throughput screening, Human pluripotent stem cells, Multiplex PCR

## Abstract

We describe a highly sensitive, quantitative, and inexpensive technique for targeted sequencing of transcript cohorts or genomic regions from thousands of bulk samples or single cells in parallel. Multiplexing is based on a simple method that produces extensive matrices of diverse DNA barcodes attached to invariant primer sets, which are all pre-selected and optimized in silico. By applying the matrices in a novel workflow named Barcode Assembly foR Targeted Sequencing (BART-Seq), we analyze developmental states of thousands of single human pluripotent stem cells, either in different maintenance media or upon Wnt/β-catenin pathway activation, which identifies the mechanisms of differentiation induction. Moreover, we apply BART-Seq to the genetic screening of breast cancer patients and identify BRCA mutations with very high precision. The processing of thousands of samples and dynamic range measurements that outperform global transcriptomics techniques makes BART-Seq first targeted sequencing technique suitable for numerous research applications.

**Electronic supplementary material:**

The online version of this article (10.1186/s13059-019-1748-6) contains supplementary material, which is available to authorized users.

## Background

Indexing of next-generation sequencing (NGS) libraries by “DNA barcodes” is crucial for economies of scale in transcriptomics studies. The pooling of indexed libraries, also known as “multiplexing”, and bioinformatics analysis of sequencing reads with indices provides a basis for quantifying the transcripts. Introduction of indices during reverse transcription is a broadly used barcoding technique for labeling thousands of different gene transcripts [1], which on the downside leads to shallow coverage per gene. In the case of analyzing thousands of single cells, using 10× genomics or Drop-Seq platforms, for example, the transcriptional information is sufficient mostly for the classification of cell types [2, 3]. Analyzing biological processes in a greater detail requires using either global indexing techniques that provide greater coverage, like SMART-Seq2 [4], but at a significantly higher cost per sample, or using targeted sequencing approaches.

Methods for targeted analysis of specific transcripts and their multiplexing from many samples are generally based on the capture of the targeted regions [5, 6], or on multiplex or 2-step PCR and ligation [7, 8]. Commercial kits such as Illumina’s Targeted RNA Expression or QiaSeq Targeted RNA Panels are based on these principles. Chief drawbacks of these methods are requirement of bulk amounts of starting material and poor dynamic range readout due to the intermittent steps of fragmentation, capture by beads or by hybridization to arrays, and nested qPCR. Additionally, padlock/molecular inversion probe (MIP)-based methods [9–11] are generally used for multiplexing very high number of loci in small number of samples. A greater accuracy for measuring gene expression is offered by microfluidic devices coupled to thermocyclers, such as the Fluidigm Biomark [12], and by probe hybridization technologies such as the Nanostring nCounter [13], MERFISH [14], FISSEQ [15], or seqFISH [16]. However, the complex workflows of these methods, and the costly operation of specialized instrumentation often prevents the analysis of thousands or even hundreds of samples, let alone application to truly massive single cell experiments.

Here, we present a novel method to serially label invariant sets of forward and reverse primers with panels of DNA barcodes, with which we generate amplicons with dual indices. We arrange the DNA barcode panels in large matrices and combine them with cDNA of bulk samples or single cells, followed by PCR and NGS. This concept of a priori sample indexing is different from the existing transcript-targeted analysis techniques, which are generally based on pre-amplification first, and indexing of the samples using DNA barcodes afterwards. The workflow, which we name Barcode Assembly foR Targeted Sequencing (BART-Seq), is inexpensive, simple, scalable, very sensitive, and accurate for omics applications using bulk samples or single cells. The relatively small number of target loci makes it readily possible to quantify gene expression. Importantly, BART-Seq can also be used for high-throughput targeted genomics, as we demonstrate in cancer patients. To make BART-Seq usable for the community, we developed a web-deployed software for designing bioinformatically optimized primers and DNA barcodes, which minimizes the sequence similarity and complementarity, hybridization to off-targets, and formation of secondary structures. Additionally, we implemented a demultiplexing pipeline to sort the amplicons to their respective samples of origin using the dual indices. Finally, we applied BART-Seq for analyzing the mechanisms of differentiation propensities of stem cells. We used human pluripotent stem cells (hPSCs) in massive sampling experiments after exposing the cells to different maintenance media and upon activation of the Wnt/β-catenin pathway at different stages of the signaling cascade. This demonstrated an effective discovery of mechanisms pertinent to cell medicines and disease modeling using BART-Seq.

## Results

**Fig. 1:**
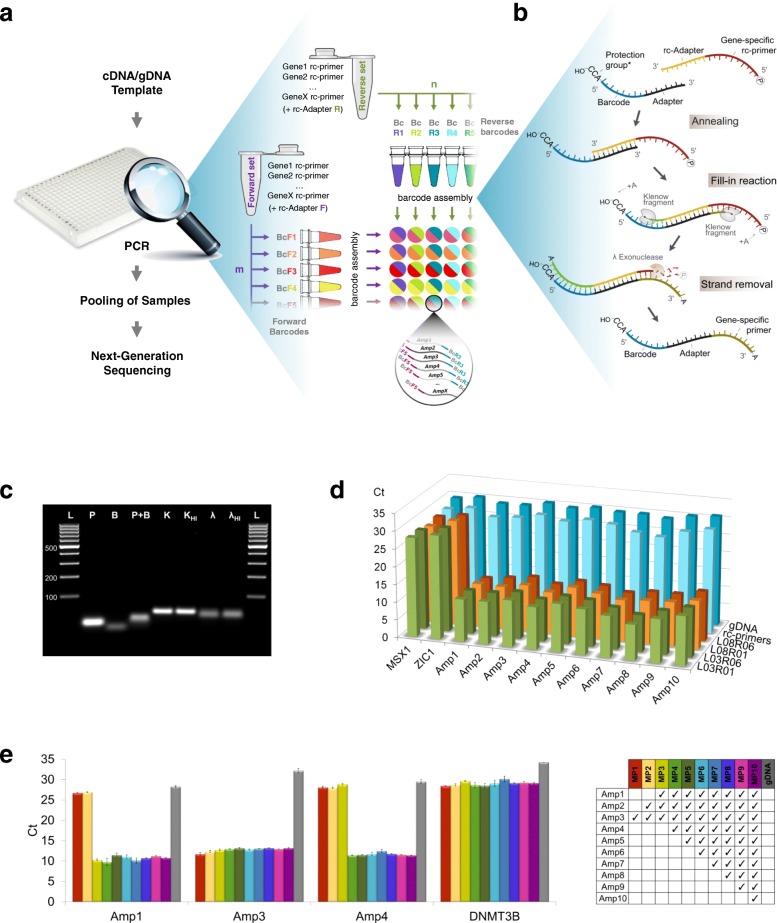
The primer-barcode assembly method for targeted amplification by PCR. **a** The principle of combinatorial indexing of a set of amplicons (Gene1-GeneX) using panels of forward (m × BcF) and reverse (n × BcR) DNA barcodes, which are used to tag invariant forward and reverse multiplexed primer sets, respectively. The predetermined targets are amplified by multiplex PCR (inset), pooled, and analyzed by NGS (any platform). **b** Primer-barcode assembly in detail: a barcode and a set of reverse complementary (rc) primers (only one is shown) are hybridized via 10-mer adapter, followed by fill-in DNA synthesis of the two strands by the Klenow fragment (an A base is frequently added to the 3′ ends). rc strands with 5′P ends are preferred substrates of λ-exo, which thereby produces barcoded single-stranded gene-specific primers. *Optional trinucleotide “protection group” that inhibits λ-exo digestion (Additional file 7: Figure S1d). **c** Gel electrophoresis demonstrating the intermediate products of the assembly process: rc primers (P) and barcodes (B) following hybridization (P + B), Klenow fill-in (K, increasing the molecular weight), and heat inactivation (K_HI_). λ-exo treatment, which creates single-stranded barcoded primers (λ, reduces the molecular weight), and heat inactivation (λ_HI_). Samples are a single barcode linked to an adapter and a single rc primer linked to an rc adapter, ran on 2.5% agarose gel with GeneRuler™ 100 bp DNA Ladder (L). **d** Co-amplification of 10 loci in *BRCA1* and *BRCA2* from gDNA using primers assembled with combinations of two forward (L03 and L08) and two reverse (R01 and R06) barcodes, and assessment of the products by qPCR using nested primers. Non-pre-amplified gDNA, non-barcoded rc primers, and non-targeted loci (*MSX1* and *ZIC1*) are negative controls. **e** Assessment of the efficiency of primer synthesis as a function of the number of multiplexed primers. Primer set size was tested for the range of 1 to 10 (increments of 1), starting with Amp3 as singleplex, with the order shown in the right pane. The concentration of the individual primers was equal in all reactions, and the barcode concentration was matched to the total primer concentration. Non-pre-amplified gDNA and the non-targeted *DNMT3B* locus were used as negative controls. Error bars represent the standard deviation of three replicates

The barcode-primer assembly method produces differentially barcoded forward and reverse primer sets for combinatorial indexing and amplification of specific transcripts by a single PCR (Fig. 1a). The assembly workflow is simple, inexpensive, lacks intermittent purification steps, and is based on oligonucleotides as the building blocks, DNA Polymerase I large (Klenow) fragment, and lambda exonuclease (λ-exo). The building blocks are eight-mer DNA barcodes coupled to ten-mer adapter sequences, and reverse complementary (rc) primer sets coupled to rc adapters. Different forward and reverse barcode panels and adapter sequences are used for the forward and reverse primer sets. The assembly protocol involves a bi-directional fill-in reaction by Klenow fragment and a unidirectional removal of the rc strand by λ-exo, which is facilitated by including a 5′-phosphate substrate in the rc primer oligonucleotides [17]. Each reaction is followed by heat inactivation of the enzymes (Fig. 1b, c).

We designed the oligonucleotide building blocks to ensure intra- and inter-primer compatibility during multiplex PCR and to minimize sample misidentification during demultiplexing. Briefly, all possible eight-mer oligonucleotides with 50–60% GC content were filtered for repeats, followed by global optimization using simulated annealing that selects barcode sequences with the lowest pairwise alignment scores. The ten-mer adapter oligonucleotides were designed the same way (listed in Additional file 1: Table S1). To generate multiplexed primer sets, we designed up to five putative primer pairs per target sequence using an implementation of Primer3 that ensures ending of the primers with a 3′ thymine (to account for template-independent addition of a single adenine (A) by Klenow fragment during primer synthesis, as illustrated in Fig. 1b). Besides the inter-primer compatibility, the efficiency of each primer set depends also on the entire set of target sequences; therefore, we used a simulated annealing approach that minimizes the secondary structure formation by evaluating pairwise folding using RNAcofold [18]. Finally, we excluded primers and barcodes that align to the human genome or transcriptome (details in “Methods” section).

To assess the barcode-primer assembly method, we first targeted specific genomic loci. We co-amplified four and six regions of the human *BRCA1* and *BRCA2* genes, respectively, using 10 pairs of multiplexed primers, which were assembled with four barcode combinations. qPCR assessment of the pre-amplified samples using nested primers, which were homologous to the assembled primers (laying downstream to the barcodes, Additional file 2: Table S2a), indicated specific enrichment of all 10 loci (Amp1-10), shown by the significantly lower Ct compared to non-pre-amplified sample, non-targeted loci, or non-barcoded rc primers (Fig. 1d, Additional file 7: Figure S1a). Importantly, increasing the number of multiplexed primers gradually from 1 to 10 did not influence the efficiency of amplification (Fig. 1e, Additional file 7: Figure S1b), indicating that the assembly method produces excess of barcoded primers for multiplexed pre-amplification of targeted loci.

**Fig. 2:**
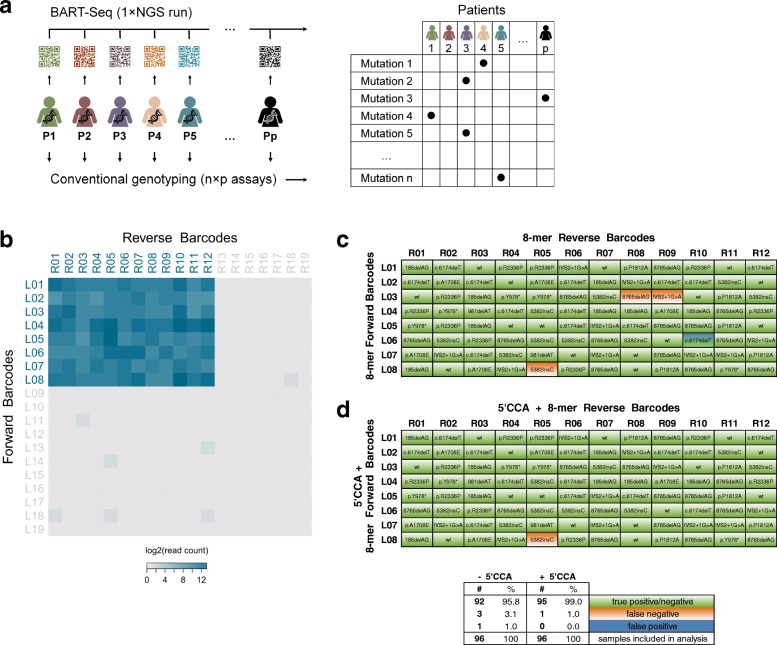
Genotyping of cancer patients using gBART-Seq. **a** Schematic representation of the application of BART-Seq for genotyping cancer patients to replace mutation-specific assays. **b** A heatmap showing the BART-Seq reads assigned to Amp4 (wild-type and mutated alleles) using gDNA of varying concentrations from 96 patients, each represented by a unique barcode combination (L01-L08 × R01-R12). L09-L19 and R13-R19 are dummy barcodes that were not used in the primer-barcode assembly. Additional amplicons are shown in Additional file 7: Figure S1c and the complete count matrices in Additional file 3: Table S3a. **c**, **d** Genotypes of 96 breast cancer patients corresponding to 10 *BRCA1* and *BRCA2* mutations. Correspondence of BART-Seq results produced using 8-mer barcodes (**c**) and barcodes with the addition of 5′CCA trinucleotide as protection group (**d**), to the known genotypes is marked by green sectors (true positives). Further details about the protection group 5′CCA is given in Additional file 7: Figure S1. A summary of patient genotyping rates for **c** and **d** is shown at the bottom

Next, we used NGS to analyze pools of barcoded amplicons that were generated by BART-Seq from cancer patient samples. *BRCA1* and *BRCA2* are breast and ovarian cancer susceptibility genes with a strong hereditary component. The Jewish Ashkenazi population is a carrier of 10 founder mutations in *BRCA1* and *2*, which reside within the loci targeted by our primer sets [19–21] (Additional file 2: Table S2a). As a template, we used genomic DNA (gDNA) obtained from 96 breast cancer patients of Jewish Ashkenazi descent that have been previously tested for a panel of 10 hereditary mutations by Sanger sequencing and other conventional assays (Fig. 2a, Additional file 2: Table S2b). We used 12 forward and 8 reverse barcodes (Additional file 1: Table S1a) for the targeted pre-amplification of the 10 *BRCA1* and *BRCA2* loci from the patients and pooled all samples for a 2 × 150 bp paired-end sequencing run using Illumina MiSeq.

Demultiplexing of the reads mapped the amplicons exclusively to the barcode combinations that were used in the experiment. Importantly, we noted only minor sorting of amplicons to 18 additional “dummy” barcodes that were not part of the experiment (Fig. 2b, Additional file 7: Figure S1c). This proved the robustness of the barcode design and demultiplexing pipeline. Comparing the genotyping results of all 1920 multiplexed alleles (spanning 10 amplicons from 96 patient samples with two alleles each) showed that 92 out of 96 patients (~ 96%) mirrored the classification of the clinical lab (Fig. 2c, Additional file 3: Table S3a).

As we serendipitously observed shortening of some of the barcodes by a few bases, which could be due to trimming of 5′ barcode ends by λ-exo during the removal the of rc strand (Fig. 1b), we sought to reduce this effect in order to further improve the classification of amplicons. To this end, we flanked the barcodes by all possible trinucleotides in order to identify the best sequences that could “protect” the 5′ of barcodes from trimming (Additional file 1: Table S1b, Additional file 2: Table S2c). Using a matrix of 5′NNN-barcoded primers tested with a constant amount of template gDNA, we observed by NGS that the trinucleotide 5′CCA had the highest frequency among all 64 combinations (Additional file 7: Figure S1d, Additional file 3: Table S3b). Repeating the patient screening using 5′CCA-barcodes, 95 out of 96 patients (99%) were classified in agreement with the results of the clinical lab (Fig. 2d, Additional file 3: Table S3c). In the misclassified sample, the expected mutation (Mut2) was detected together with an unexpected mutation (Mut7), indicating that the misclassification might be due to sample cross-contamination. Collectively, these developments created a robust workflow for targeted sequencing in genomics studies, which we named gBART-Seq.

**Fig. 3:**
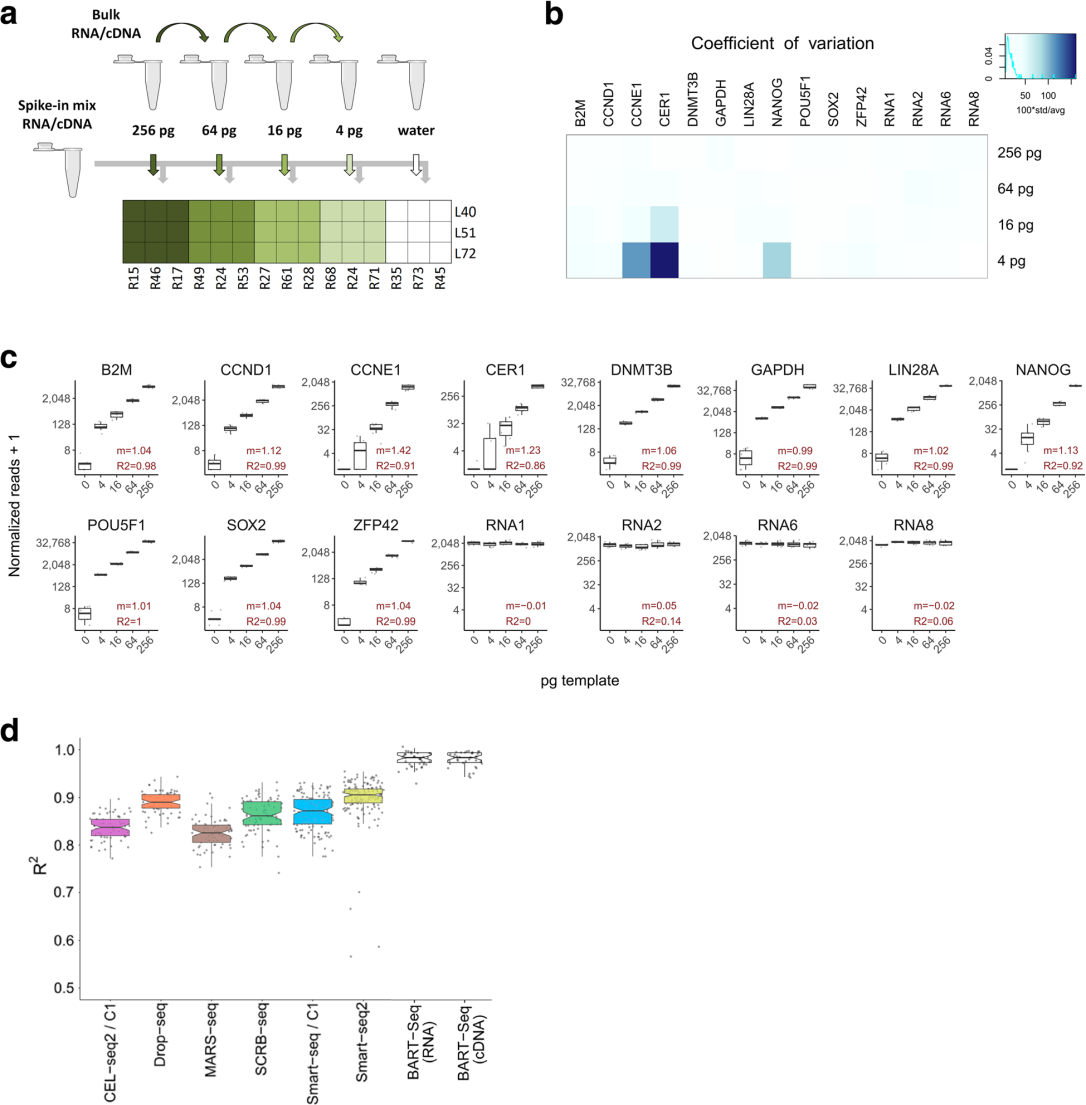
Transcript quantification using rBART-Seq. **a** Fourfold serial dilutions of bulk RNA isolated from hPSCs [22] were combined with constant amount of spike-in RNA mixture, aliquoted into nine replicate wells (4–256 pg/well), and reverse transcribed, each of which was then indexed with a different barcode combination during PCR. Water mixed with spike-ins was included as a negative control. The experiment was repeated by reverse transcribing the bulk RNA and spike-in mixture separately and combining respective bulk cDNA dilutions with spike-in mix cDNA (Additional file 7: Figure S2). **b** The coefficient of variation of the normalized reads obtained from RNA dilution samples in **a** calculated for the groups of nine samples receiving identical template concentration, but different barcode combinations. The average was less than 25%. **c** Boxplots showing normalized read counts assigned to 11 transcripts and three RNA spike-ins, plotted against template concentration. Slopes (*m*) were close to 1 for the majority of the samples, and coefficients of determination (*R*^2^) were higher than 0.96 on average, in the linear regression models calculated for the 4–256 pg sample groups. **d** A plot based on Ziegenhain et al. [1], displaying the adjusted *R*^2^ values of linear regression models calculated using ERCC spike-in expression values obtained using different global transcriptomics methods as indicated. Corresponding BART-Seq values were obtained by calculating linear regression models using the average read counts of 11 genes across the experiment to model the reads observed in individual samples. *R*^2^ values had a median of 0.98 in the BART-Seq experiments

Because BART-Seq is based on a single PCR workflow that circumvents intermittent steps of fragmentation, hybridization, or ligation, which hinder quantitative analysis, we postulated that the method is suitable for targeted sequencing and quantification of RNAs. To create the rBART-Seq workflow (for RNA), we produced sets of forward and reverse primers that target 11 human pluripotency and housekeeping gene transcripts (five exon spanning), as well as four exogenous RNA spike-in molecules, which we validated by nested qPCR (Table 1, Additional file 7: Figure S2a, b, Additional file 2: Table S2d). We first created a dilution series of purified RNA from hPSCs, and combined the samples with fixed amounts of the four spike-in RNAs for normalization purposes (Fig. 3a). Importantly, although we analyzed samples of picogram concentrations, the variations among the equimolar replicates tagged with different barcodes were very low, and the correlation between the template RNA concentration and gene reads was very high (both for normalized and raw reads; Fig. 3b, c; Additional file 7: Figure S2c-g, Additional file 4). The only exceptions were a few genes in the lower end of the dilution series (e.g., 4 pg), such as *CER1*, which is marginally expressed in undifferentiated cells. These data also demonstrated the negligible effect of diverse barcodes on the read counts. When we compared the correlation scores produced by BART-Seq with global single-cell sequencing techniques reviewed by Ziegenhain et al. [1], we noted that BART-Seq exhibits outstanding accuracy (Fig. 3d).

**Table 1:** List of targeted genes in transcriptomics experiments

Panel	Number of targets	List of targets
Pluripotency	15	Housekeeping: *B2M*, *GAPDH*; pluripotency: *NANOG*, *POU5F1/OCT4*, *SOX2*, *LIN28A*, *DNMT3B*, *ZFP42/REX1*; cell cycle: *CCND1*, *CCNE1*; control: *CER1*, 4 RNA spike-ins
Differentiation (Wnt/β-catenin)	22	Lateral mesoderm: *EOMES*, *HAND1*, *MESP1*; paraxial/(pre-)somitic mesoderm: *CDX2*, *HOXA1*, *MSGN1*, *PAX3*, *TBX6*; neural crest: *ZIC1*, *MSX1*; pan-primitive streak: *EVX1*, *GSC*, *MIXL1*, *T*; anterior primitive streak: *NODAL*; endoderm: *FOXA2*, *SOX17*; control: *GAPDH*, *NANOG*, 3 RNA spike-ins

**Fig. 4:**
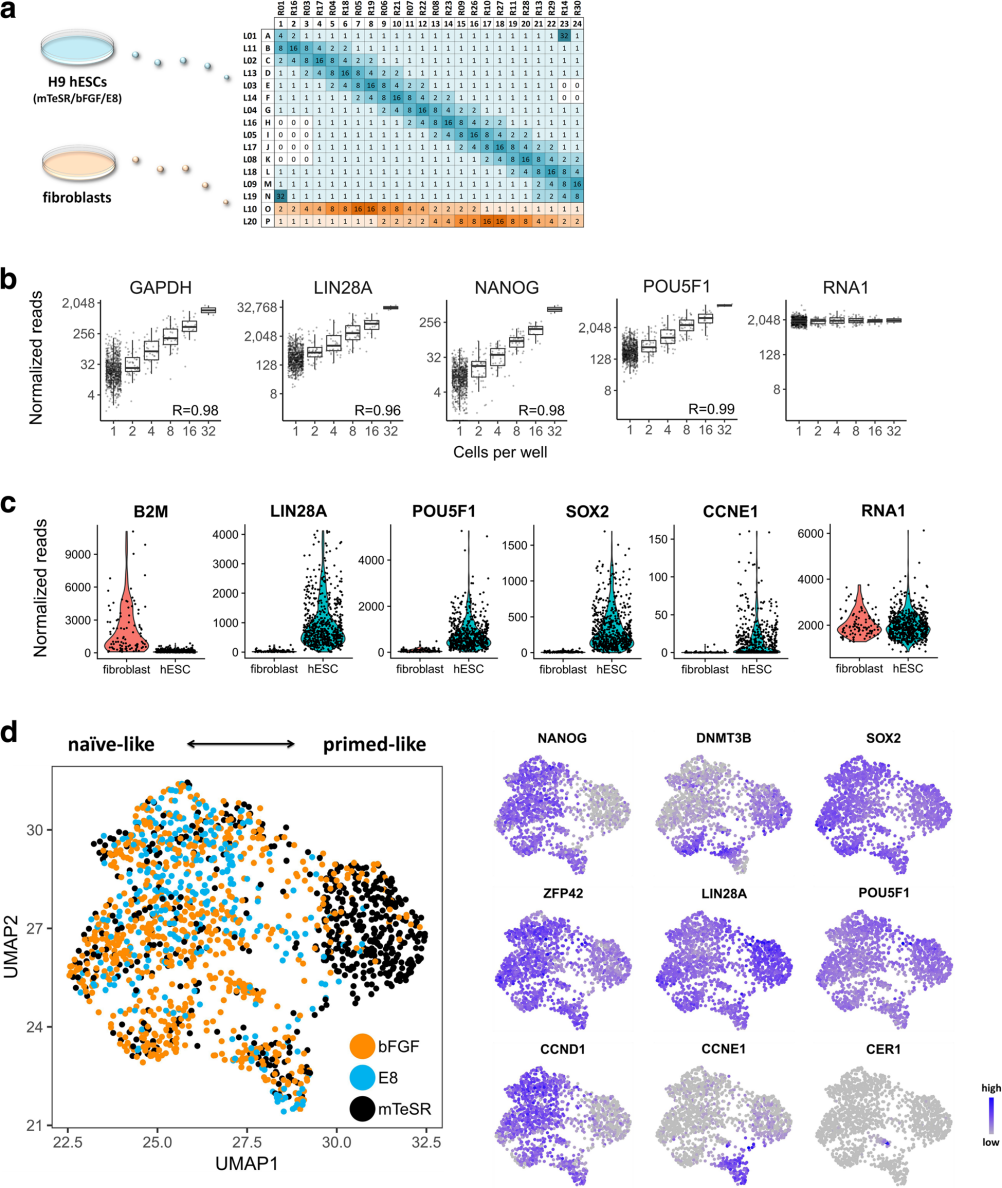
Transcriptional heterogeneity of single hESCs. **a** Part of the barcode matrix used for the analysis of single (1) and multiple (2, 4, 8, 16, 32) hESCs maintained by different media (mTeSR™1, KSR-bFGF, and E8) and BJ fibroblasts. Negative controls were wells not receiving sorted cells (0). Prior to sorting, all wells (including negative controls) were pre-filled with 2 μl of RT mixture containing fixed concentrations of four RNA spike-ins. Over 4500 wells representing two biological replicates were analyzed as two libraries and sequenced using Illumina NextSeq for a total of 23.5 million processed paired reads. **b** Normalized read counts of selected genes plotted against the number of cells sorted per well (*n* = 858 samples from KSR-bFGF medium are shown). Correlation coefficients (*R*) between the cell counts and the median of corresponding reads are shown. **c** Violin plots illustrating the expression of a subset of genes by hESCs and fibroblasts. Samples include single cells and calculated one-cell values of multi-cell wells. Higher *B2M* expression by fibroblasts was noted [23], while pluripotency and cell cycle genes had notably higher expression in the hESCs. RNA1 represent the spike-ins. **d** UMAP projection of single hESCs (*n* = 1550) treated with three media (black dot, mTeSR; orange dot, bFGF; light blue dot, E8), with respect to 11 genes. Expression of some of the genes underlying the distribution is plotted on the right. All results are based on two biological replicates, and plots for the rest of the genes (and conditions) for **b** and **d** are shown in Additional file 7: Figure S4

We next applied rBART-Seq for direct measurements in single cells and asked whether it is possible to detect subtle changes in the expression of the core pluripotency network of transcription factors when hPSCs are treated by different maintenance media. We sorted over 4500 wells with human embryonic stem cells (hESCs) that were cultured in mTeSR™1, KSR-bFGF, or E8 media, and in parallel BJ fibroblasts, directly into reverse transcription (RT) reaction mix that contained four RNA spike-ins (Fig. 4a). We normalized the data using spike-ins and omitted samples with low signals that were operationally defined as empty wells (Additional file 7: Figures S3, S4a). We noted a very high correlation between the number of sorted cells per well and the corresponding reads and the highest transcriptional variation in single cells (Fig. 4b, Additional file 7: Figure S4b, Additional file 5: Table S5). Moreover, the expression profiles of hESCs and fibroblasts were significantly different (Fig. 4c), although many fibroblasts were mapped with some reads of pluripotency genes.

We subsequently discovered that this was due to the index switching [24], spreading primarily from the wells with the highest concentration of RNA from hESCs. We later minimized this effect by increasing the diversity of the samples in the flow cell (e.g., using PhiX control or co-sequencing with non-BART-Seq libraries), and noted that this effect became marginal in those experiments (e.g., 0 pg samples in Fig. 3c). Taken together, these data show that rBART-Seq can be used for directly analyzing gene expression in numerous single cells and produce results with a broad dynamic range.

In accordance, we applied non-linear dimensionality reduction (UMAP) to analyze the single hESCs that were grown with the three maintenance media. This revealed two major subpopulations exhibiting ground state-like —*NANOG*^HIGH^ *ZFP42* (*REX1*)^HIGH^— and primed-like —*LIN28A*^HIGH^ *DNMT3B*^HIGH^— phenotypes [25–28] (Fig. 4d). Remarkably, mTeSR™1-treated cells were located primarily in the primed-like cluster, while the majority of the E8-treated cells were located in the ground state-like (naïve) cluster, suggesting that these growth conditions shift hESCs along the pluripotency axis. This indicates that preferences to use mTeSR™1 over E8, or vice versa, as a starting point for differentiation may depend on how well different protocols are tuned to the respective states of pluripotency, for example, in the case of cardiomyocyte differentiation [29–31].

**Fig. 5:**
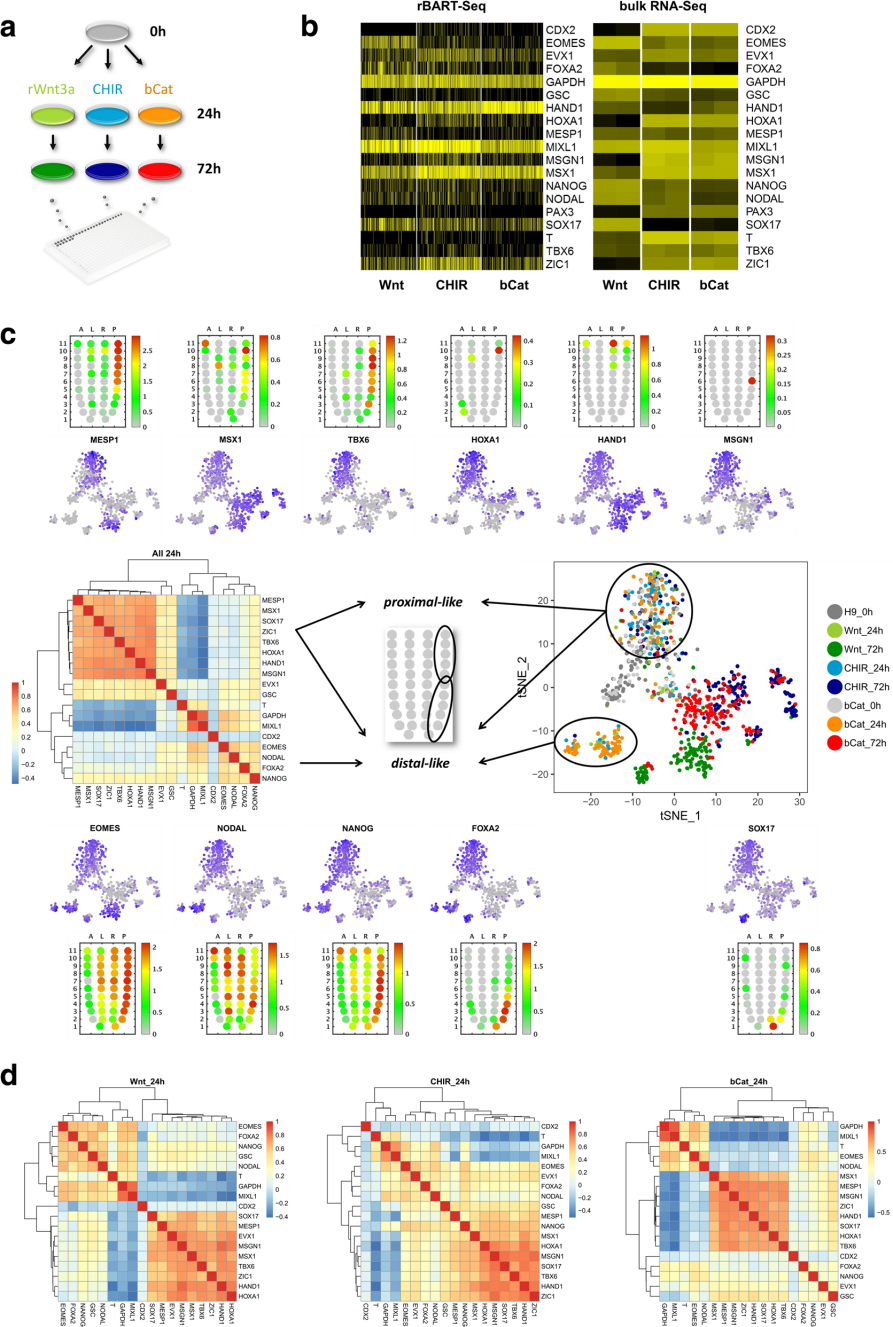
Cell populations emerging upon stimulation of the Wnt/β-catenin pathway at different stages of the cascade. **a** A 72-h time course differentiation experiment of hESCs that were treated by recombinant Wnt3a, CHIR99021 (CHIR), or with doxycycline (dox) to induce the expression of transgenic β-cateninΔN90. Single cells were sampled at 0, 24, and 72 h for rBART-Seq analysis. A total of 4324 cells from three biological replicates were analyzed in a single NextSeq Mid Output run. **b** Heatmaps of the 19 genes analyzed by rBART-Seq (72 h, left) and TPM values (transcripts per million) of the same genes analyzed by bulk RNA-Seq, based on two independent replicates per condition (right). **c** A heatmap of the pairwise gene correlations calculated based on single cells at 24 h from the three treatments (left) and two-dimensional representation (tSNE) of the single cells sampled at 0, 24, and 72 h from all treatments, based on the expression of 19 genes (right). Expression of selected genes underlying the tSNE plot is shown in the upper and lower panels. The corn plots were derived from the iTranscriptome database [36] representing the locations of expression of the genes in epiblast stage mouse embryos (E6.5-E7.5). **d** Heatmaps of the pairwise gene correlations at 24 h for each of the treatments separately. Data presented in this figure represent one of the replicates. Rest of the genes and data from another replicate are shown in Additional file 7: Figure S5. Count matrices of all three biological replicates are available as Additional file 6: Table S6

Finally, we applied rBART-Seq to test the claim that GSK3β inhibitors mimic the ligands of the Wnt/β-catenin pathway in the differentiation and maintenance of different types of stem cells [32–34]. We treated hESCs by recombinant Wnt3a (rWnt3a) or the broadly used small molecule inhibitor of GSK3, CHIR99021. In addition, we integrated doxycycline (Dox)-inducible constitutively active β-catenin (ΔN90) to hESCs in order to test if, as speculated, CHIR99021 exerts its effect only by stabilizing β-catenin [35]. We sorted the cells before and following 24 and 72 h of stimulation and applied the rBART-Seq for the analysis of 22 markers of early gastrulation, housekeeping genes, and 3 RNA spike-ins, which we also validated by nested qPCR (Table 1, Fig. 5a, Additional file 7: Figure S5a). When we inspected the same panel of genes in the global sequencing of bulk RNA following 72 h of stimulation, we observed a striking similarity between β-cateninΔN90 and CHIR99021, but differences to Wnt3a treatment. Analysis of the rBART-Seq single-cell data showed remarkable resemblance to the global RNA-Seq results despite a significant degree of cellular heterogeneity (Fig. 5b**,** Additional file 6: Table S6). Moreover, pairwise gene correlation analysis after 24 h of stimulation revealed two clusters exhibiting *MESP1*, *MSX1*, *SOX17*, *ZIC1*, *TBX6*, *HOXA1*, *HAND1*, *MSGN1*, and *NANOG*, *NODAL*, *EOMES*, *FOXA2* gene signatures (Fig. 5c, left). This reflected the emergence of two cell subpopulations, as shown by dimensionality reduction (tSNE) analysis (Fig. 5c, right), which likely correspond to the proximal and the distal region of the embryo, respectively, as indicated by the topology of expression of the orthologous genes in the mouse embryo [36]. Pan-primitive streak markers *GSC*, *EVX1*, and *MIXL1* correlated with both groups, while *MIXL1* was expressed at a higher level in the distal-like group (Fig. 5c, d; Additional file 7: Figure S5b, c). With respect to the influence of different stimulations of the Wnt/β-catenin pathway, the distinct clusters were apparent after 72 h, and Wnt3a treatment produced definitive endoderm-like and lateral plate mesoderm-like cells, with *FOXA2*^HIGH^ *SOX17*^HIGH^ and *HAND1*^HIGH^ *MESP1*^HIGH^ *EOMES*^HIGH^ profiles, respectively. The latter population dominated the Wnt3a progeny in the replicate experiments (Fig. 5c). Taken together, we concluded that CHIR99021 limits the diversity of primitive streak-like progeny that differentiates from hESCs compared to the ligand of the pathway Wnt3a, an effect that was also validated using constitutively active β-catenin.

## Discussion

Massive sequencing of defined sets of transcripts could be highly useful for numerous studies that involve analysis of vast arrays of samples in parallel. The application areas include probing of mechanisms; single-cell analysis; validating and complementing results obtained by genome-wide approaches, such as the Human Cell Atlas Project [37]; and screening in genome engineering, drug development, and toxicology assays. To become truly impactful, a targeted sequencing method should enable serial and straightforward production of libraries from individual samples, be cost competitive compared to genome-wide approaches, and involve simple bioinformatics analysis. It should also be sensitive and quantitative as genome-wide transcriptomics techniques or have better performance. We show here that BART-Seq is in fact the first transcript-targeted sequencing approach that fulfills these criteria.

BART-Seq overcomes important limitations of other targeted sequencing approaches. First, the workflow does not include intermittent steps of template purification that are generally incompatible with gene expression analysis as the case for other methods [7, 8]. Importantly, the entire BART-Seq process, from primer assembly to count matrices, can be completed within 5 days. Second, BART-Seq creates sequence data, while other methods such as MERFISH or seqFISH infer it from hybridization of probes [14, 16], which could limit the discovery of sequence variants and may require further validation. Third, BART-Seq is an inexpensive technique compared to other targeted and global approaches. Our estimate for the full cost of analysis per sample, e.g., one well of a 384-well plate, consisting of a single-cell or bulk gDNA/cDNA, is approximately 1 US dollar. Compared to the global transcriptomics techniques, this places BART-Seq just above Drop-Seq which is lowest in terms of cost per sample [1]. Fourth, compared to Drop-Seq, 10×, and other methods, BART-Seq can be used to analyze a broader range of target RNAs in single cells, including non-polyadenylated lncRNAs, simply by the use of specific primer sets or random hexamers. For this reason, the method is also readily compatible with genomics studies as we show here (Fig. 2). Therefore, it could be used as an inexpensive and technically straightforward alternative to protocols involving nested PCR [7, 8, 38], gDNA circularization [38, 39], or MIPs [11] in genomic studies. We have not yet combined the use of unique molecular identifiers (UMIs) [40] with BART-Seq, which may be important in some applications for counting the absolute number of transcripts.

BART-Seq can expand the segment of targeted transcriptomics, which has not been fully exploited yet, especially in the high-throughput analysis of single cells. Using simple bioinformatics that sorts tens of thousands of amplicons that were indexed by BART-Seq, we gained important insights into the mechanisms that regulate the differentiation of hPSCs. We found that pluripotency is held at different depths when frequently used maintenance media are applied to hPSCs, a phenomenon that could explain the many cases of matching between certain maintenance media and differentiation protocols [29–31]. Moreover, we showed that the application of a small molecule that is widely considered an agonist of the Wnt/β-catenin pathway, in fact, reduces the repertoire of primitive streak-like progenitors that differentiate from hPSCs. Importantly, these experiments demonstrate that a key feature of BART-Seq is multivariable analysis, in this case of cells that were subjected to different treatments and were sampled at different time points, which can be achieved by simply increasing the size of the barcode panels (Fig. 1a). If we were to analyze the same cohort of samples as we did here by Drop-Seq or 10× techniques, for example, the analysis costs would have been drastically higher due to the use of different preparation kits for every iteration of time point, treatment, and biological replicate [2, 3]. This indicates that BART-Seq is particularly useful for kinetics studies, screens, and for linking phenotypes, e.g., fluorescent marker intensity, to the expression of transcripts and/or genotypes, a level of information that is lost with techniques that utilize pools of cells and barcoded droplets [2, 3, 41, 42].

## Methods

### Design of barcode panels

All possible 8-mer (barcode) and 10-mer (adapter) oligonucleotides of 50–60% GC content were computed omitting sequences with one, two, or three nucleotide repeats. All pairwise global alignment scores were computed separately for barcodes and adapters using pariwise2 from Biopython package. Whenever comparing two barcodes in all forward and reverse combinations, the maximal alignment scores were used for further analysis. Next, a global optimization heuristic (simulated annealing) was implemented to efficiently identify a set of highly unique sequences in terms of likelihood that mutations (exchange, deletion, insertion) might lead to a conversion into another sequence within the set. A random initial set of sequences was either shrunk (with 10% probability), altered by randomly exchanging sequences (36% probability), or randomly increased (54% probability). Changes were accepted if the new sum of alignment scores was lower or by change whenever exp(−Δsumscore÷*T*) was lower than another random number. This simulated annealing algorithm scanned temperatures *T* from 10,000 to 0 along 300 cooling iterations to reach a global optimum. The resulting sets were randomly divided into forward and reverse barcodes and adapters. Next, the 3′ of the forward and reverse adapters were ligated in silico to the sequences of the forward and reverse barcode sets, respectively. Finally, BLAST was used to accept 18 nt sequences without any identified hit in the human genome (for barcodes version 1, used for genomics) and transcriptome (for barcodes version 2, used for transcriptomics) as outlined in Additional file 1: Table S1.

### Primer design and optimization

Primers were designed to amplify roughly 80–250 bp amplicons in locations where an adenine (A) base exists at the 3′ position of the final primer sequence after barcode assembly. This was done because the DNA Polymerase I large (Klenow) fragment frequently adds a template-independent A base to the 3′ of the newly synthesized strand. Primer3 was used with default settings, but with modified internal primer predictions such that it enforces the primer’s 3′ to end with a T nucleotide. For each template, up to five forward and reverse primer pairs were predicted. Each primer pair set was compared against the human genome using the blastn command from the blast+ package with the parameters -reward 1 -gapopen 5 -gapextend 5. Using our web-based software, the user can set the number of hits allowed for further processing. Next, given the predefined set of barcodes, adapters, and 1–5 predicted primer pairs per loci, an in silico ligation step was performed to generate all possible primer-adapter-barcode combinations. Hereby, matching forward and reverse primers defined one amplicon. To minimize the probability of forming stable dimers, we calculated the all-against-all minimal free energy (including all reverse complements) using the RNAcofold command from the ViennaRNA package version 2.1.8 with the parameters --noPS --noLP -P dna_mathews2004.par. Low predicted minimum free energy correlates to a high probability of forming a stable dimer. A simulated annealing was implemented to identify optimal combinations of each primer pair per locus, thereby taking barcode and adapter sequences into account. During optimization, the minimal value of free energy of the forward or reverse complement sequence was used for determining the probability of forming stable primer dimers. Per amplicon and gene, we started with a random initial set of primers. We proceeded to either randomly alter it (with 80% probability) or randomly exchanged amplicons if there were several amplicons available for a gene. In each step, the random change was accepted if the new sum of minimal free energies (*mfe*) is lower than in the last or randomly if exp(−Δmfe÷*T*) was lower than a uniformly drawn random number. We scanned over temperatures *T* from 15,000 to 0 along 500 cooling iterations. Finally, we reversed the primer sequences and linked 3′ to the reverse sequence of the respective forward or reverse adapter sequences. The primer prediction implementation is a Python-based web front end that is available online at: http://icb-bar.helmholtz-muenchen.de, of which we made the code freely available (see “Availability of data and materials” section).

### Design of primer sets

Primer sets targeting 10 specific mutations in *BRCA1* and *BRCA2* genes [19–21] were designed based on the human genome reference hg19 (Additional file 2: Table S2a, c). Amplicon size was in the range of 75 to 248 nt to ensure detection by 2 × 150 bp paired-end sequencing. Pluripotency primer set was designed based on the analysis of publicly available RNA-Seq datasets of hESCs via NCBI-GEO from H9, H7, and HD291 cells (GSM602289, GSM1163070, GSM1163071, GSM1163072, GSM1704789, GSM1273672, GSM1327339), and own datasets. The target regions were selected for differentiation primer set using bulk RNA-Seq data produced by stimulation of hESCs by Wnt3a or CHIR99021 for 72 h. RNA-Seq reads were mapped to the genome reference hg38 using CLC Genomics Workbench (version 8.5.1) using mismatch cost: 2, insertion cost: 3, and deletion cost: 3. The regions mapped with a significantly high number of reads overlapping in the majority of the samples were used for primer design. The complete sequences of RNA spike-ins EC2 (RNA1), EC12 (RNA2), EC13 (RNA6), and EC5 (RNA8) were used as target regions (Ambion, AM1780).

### Cell culture

Undifferentiated hESCs (H9 line) were maintained on Matrigel™ (Corning)-coated plates in mTeSR™1 medium (Stem Cell Technologies) in 5% (*v*/*v*) O_2_. Cells were passaged as clumps using 2 mg/ml solution of Collagenase Type IV prepared in DMEM F-12 (both from Thermo Fisher Scientific).

#### Growth media comparison

Cells were split and maintained for five passages in mTeSR™1, E8 (on Matrigel™), and KSR-bFGF media (on CD1-irradiated mouse embryonic fibroblasts) in parallel. E8 medium was prepared as described by Chen et al. [43] and KSR-bFGF media as described by Krendl et al. [44]. Newborn human BJ fibroblasts (ATCC®) were cultured in DMEM high glucose (Thermo Fisher Scientific), supplemented with 1% GlutaMAX (Life Technologies), NEAA (Thermo Fisher Scientific), and 10% HyClone™ Fetal Bovine Serum (GE Healthcare).

#### Wnt/β-catenin pathway activation

hESCs and hESC line modified with doxycycline-inducible β-catenin (constitutively active form ΔN90) were maintained on Matrigel™-coated plates in mTeSR™1 medium with 25 μg/ml Hygromycin B (Thermo Fisher) in the case of β-cateninΔN90 line. For time course stimulations, the cells were dissociated to single-cell suspension with Accutase (Sigma) and seeded into 12-well plates at 2.5 × 10^5^ cells per well in the presence of 10 μM Y-27632 (R&D Systems). The next day, the medium was changed to RPMI-1640 with l-glutamine supplemented with 1× non-essential amino acids and 1× B27 supplement without insulin (all from Life Technologies). Ligands were as follows: 10 μM CHIR99021 (Tocris) and 240 ng/ml recombinant Wnt3a (gift from Derk ten Berge, Erasmus University Medical Centre, Rotterdam). β-catenin expression was induced by adding 1 μg/ml doxycycline (Clontech). The medium and ligands were freshly re-added every 24 h.

### Single-cell sorting and cDNA synthesis

#### Sorting

hESCs were dissociated using Accutase (Sigma), and cells maintained in KSR-bFGF on MEFs were collected as clumps using Collagenase Type IV prior to Accutase treatment. Newborn human BJ fibroblasts were dissociated using Trypsin-EDTA 0.25% (Gibco). For sorting, the cells were resuspended in 1 ml of FACS buffer (4% FBS and 5 μM EDTA in PBS), filtered through a 0.2-μm nylon mesh, and single live cells (propidium iodide negative) were sorted into the 384-well plates (1–32 cells for medium comparison, and single cells for Wnt pathway activation) pre-filled with 2 μl reverse transcription mixture, using Aria III sorter (BD Biosciences).

#### cDNA synthesis

Reverse transcription mixture (RT mix) was prepared using SuperScript™ III First-Strand Synthesis System (Invitrogen) with reverse transcriptase at a final concentration of 2.5 U/μl (nuclease-free water) and Oligo-dT primers (2.5 μM). RNA spike-ins were included in the RT mix (experiment-specific concentrations). Following sorting, plates were sealed with adhesive foils, placed immediately on dry ice for 2 min, and stored at − 20 °C. Plates were thawed at room temperature, and the reverse transcription was performed using the thermocycler program: 50 °C for 50 min and 85 °C for 5 min; RNaseH was not used.

#### Bulk RNA isolation

Total RNA was extracted using RNeasy Mini Kit (QIAGEN).

### Barcode assembly

#### Klenow fill-in reaction

Unit reaction mixture was prepared in nuclease-free water by combining 1× React® 2 Buffer (Invitrogen), 0.267 mM dNTPs, 2.5 μM multiplexed rc primer mix, 2.5 μM barcode, and 0.0167 U/μl DNA Polymerase I large (Klenow) fragment (Invitrogen). The reaction was incubated at 25 °C for 1 h. Individual rc primers were used at a 0.025-μM final concentration, and barcode concentrations were matched to the total concentration of rc primers (incubation time of 2 h was also applicable). The enzyme was heat inactivated at 80 °C for 10 min.

#### Reverse complementary strand removal by lambda exonuclease

Products of the fill-in reaction were directly diluted as 2/3 volume ratio in the lambda reaction mixture containing 1× reaction buffer and 0.33 U/μl lambda exonuclease (New England Biolabs) and incubated at 37 °C for 30 min (incubation time of 1 h is also applicable). The enzyme was heat inactivated at 80 °C for 10 min.

#### Pre-amplification PCR

PCR reactions (10 μl total) consisted of 2.5 μl (0.5× final) Platinum® Multiplex PCR Master Mix (Applied Biosystems), 1.8 μl 25 mM MgCl_2_ (4.5 mM final), 1.5 μl forward lambda reaction product (non-purified), 1.5 μl reverse lambda reaction product (non-purified), 2 μl cDNA, and 0.7 μl nuclease-free water (not DEPC-treated). The reaction cycle profile was as follows: initial denaturation at 95 °C for 5 min; 22 cycles of 95 °C for 30 s, 60 °C for 3 min, 72 °C for 60 s; and final extension at 68 °C for 10 min. Unit PCR reaction of genotyping assays was 20 μl, with the same concentration of reagents, and 18 cycles of PCR. Unit PCR reaction of transcriptomics experiments was 10 μl, with cycle numbers between 16 and 22.

### qPCR and melting curve analysis

qPCR analyses were performed using nested primers, which are homologous to the barcode-assembled primers, excluding the barcode and the adapter regions (Additional file 2: Table S2). Unit reaction (10 μl total) consisted of 5 μl (1× final) Power SYBR™ Green PCR Master Mix (Applied Biosystems), 1 μl pre-amplification PCR product, 1 μl forward and reverse nested primers mix (each 0.2 μM final), and 3 μl nuclease-free water (not DEPC-treated). The reaction cycle profile was as follows: initial denaturation at 95 °C for 10 min followed by 35–40 cycles of 95 °C for 15 s and 60 °C for 1 min. Melting curve analysis was done by heating the amplicons from 60 to 95 °C, incrementing 0.05 °C/s. All the reactions were run as three replicates.

### Next-generation sequencing

#### Sample pooling and purification

PCR products were pooled in nuclease-free falcon tubes (Ambion), mixed with 0.1 volume 3 M NaOAc (pH 5.5) (Ambion) and 2.5 volume 100% ethanol (molecular biology grade), and kept at − 20 °C overnight for precipitation. Samples were centrifuged at 4000*g* for 30 min in a centrifuge pre-cooled to 4 °C. The supernatant was discarded, and the samples were washed once with 500 μl ice-cold 70% ethanol. Tubes were centrifuged at 4000*g* for 2 min (4 °C), and the remaining supernatant was pipetted out. The pellet was air dried for 2–3 min and re-suspended in 200–400 μl nuclease-free water. Prior to library preparation, double-sided size selection was performed using Agencourt AMPure XP beads (Beckman Coulter). 0.5× and 1.5× bead to DNA ratio was used for upper and lower size limits, respectively.

#### RNA-Seq library preparation and sequencing

Libraries were prepared using NEBNext® Multiplex Oligos for Illumina® (New England Biolabs, E7335), and the protocol was based on NEBNext® ChIP-Seq Library Prep Master Mix Set for Illumina® (New England Biolabs, E6240) with the following modifications: end repair was performed using 1 μl NEBNext End Repair Enzyme Mix in 50 μl final reaction. PCR enrichment included 1 μl index and 1 μl universal primers in 50 μl final reaction. The enrichment PCR cycle profile was as follows: initial denaturation at 98 °C for 30 s; 10–15 cycles of 98 °C for 10 s, 65 °C for 30 s, 72 °C for 30 s; and final extension at 72 °C for 5 min. Fifteen, 15, 12, and 10 cycles of PCR enrichment was applied for genotyping, bulk dilution, media comparison, and mesoderm experiments, respectively. Beads to DNA ratios for purification steps using AMPure XP beads were adjusted according to the expected maximum and minimum amplicon size of the individual libraries. Libraries were evaluated using Agilent 2100 Bioanalyzer by High Sensitivity DNA Kit (Agilent) and quantified using Qubit® 2.0 Fluorometer by Qubit® dsDNA HS Assay Kit (Invitrogen), and by Safire II Microplate Reader (Tecan) using Quant-iT™ PicoGreen™ dsDNA Assay Kit (Invitrogen). Libraries were sequenced (paired-end) on Illumina MiSeq using MiSeq® Reagent Kit v2 (300 cycles) or Illumina NextSeq 500 using NSQ® 500/550 Mid Output Kit v2 (300 cycles). Ten percent PhiX control (Illumina, #FC-110-3001) was included in the sequencing runs as a measure against index switching [24] for low-diversity libraries like BART-Seq.

### Demultiplexing of RNA-Seq reads to count matrices

To trace the origins of reads back to the samples, a pipeline that demultiplexed the reads and counted them while accounting for sequencing errors was implemented. FastQC software was used to create quality reports for manual inspection [45]. Given the acceptable quality, Snakemake workflow engine [46] was used for automatic or step-by-step analysis of raw reads, sets of primers, linkers, barcodes, and expected amplicons. This started by trimming the read ends according to quality using Sickle [47], then a list of possible single nucleotide-mutated variants per barcode, excluding the ones shared with other barcodes, was created. Using the algorithm of Aho and Corasick [48], this list efficiently assigned barcodes to all reads while allowing at most one unambiguous mismatch. We also annotate the reads with several boolean criteria for statistical analysis of libraries. This included the information if the read contained only a primer, multiple (or no) barcodes, if the barcode contained a mismatch or if the read contained bases before the protection group. We aligned the longer amplicons to the reads using HISAT2 [49]. The final step of the pipeline is to summarize the results. Heatmaps for each library were created per amplicon using the forward and reverse barcodes as a coordinate system, and a spreadsheet file containing the aforementioned read statistics as well as count matrices was generated. The pipeline was also made available as described in “Availability of data and materials” section.

### Classification of *BRCA* mutations

To classify the amplicons corresponding to mutations 1–10, we generated read count per patient for both wild-type and mutation alleles (identified by top blast hit per read) and assigned the mutation type with the highest mutation read count. Read count ratios of mutation to wild-type allele per sample were computed and accepted as the mutation for ratios > 0.20.

### Analysis of protection group

For the analysis of 5′ protection groups, we identified barcodes using BLAT [50], a BLAST-like alignment tool, with options -minScore=0 -minIdentity=95 allowing for one base mismatch at most. This was necessary to screen all possible protection groups. For each detected wild-type or mutant allele, we calculated the frequency of 64 trinucleotides for each forward and reverse barcode. Then, summing the frequencies up across all the alleles, we obtained the total frequency of each trinucleotide per barcode.

### Data correction and normalization

#### Correction of RNA spike-in reads

First, all wells with extreme outlier spike-in reads were manually removed after inspecting the heatmaps of raw read counts (i.e., if exhibiting hundreds of folds higher/lower reads than the average). Per gene, samples exhibiting extremely low barcode-gene combinations were removed. Then, per spike-in, two-sided *t* test (default parameters, R version 3.5.2) was performed for each barcode against the rest of the barcodes of the same type (i.e., forward or reverse), using the data between the 5th and 95th percentiles for both groups. Barcode-spike-in combinations with *p* values lower than the set threshold were replaced with the median of the rest of the barcodes.

#### Normalization of the data

Scaling factors (RNA_*x*_) were calculated using spike-ins (left) or spike-ins and genes together (right) as follows:

$$ {\mathrm{RNA}}_{\mathrm{x}}={2}^{\left(\frac{1}{n}{\sum}_1^n{\log}_2\left({\mathrm{spike}}_n+1\right)\right)}\ \mathrm{or}\ {\mathrm{RNA}}_x={2}^{\left(\frac{1}{n}{\sum}_1^n{\log}_2\left({\mathrm{gene}}_n+1\right)\right)} $$

Wells were removed if the scaling factor was tenfold lower or higher than the median, to prevent overcorrection. Then, the factors were median-centered via division to preserve the read count magnitudes. Finally, raw read counts of the transcripts were divided by the scaling factors (Additional file 7: Figure S3). The corresponding script is available at the Github (see the “Availability of data and materials” section). An alternative and more precise method for normalizing the data based on the correction of spike-ins using negative binomial generalized linear modeling is also provided in the same repository.

### Well filtering in single-cell experiments

Wells sorted with single cells were operationally defined as “empty” if the ratio of the sum of the spike-in reads to the total reads per sample (normalized and log-transformed) was same or higher than the negative controls (into which no cells were sorted) (negative control wells received some reads due to index switching). Samples representing the wells sorted with multiple cells were filtered based on the calculated one-cell values of the genes. Filtering the samples sorted with two cells or more, i.e., “doublets,” was done by placing a threshold estimated based on the bimodal distribution of the sum of the genes (log2-transformed) (Additional file 7: Figure S4a). Only housekeeping genes were used for filtering fibroblasts.

### Analysis of gene expression

Gene expression analyses were done using custom scripts or Seurat package in R (version 2.3.4), based on normalized and log2-transformed read counts. Linear regression models were calculated using lm function (default parameters, R version 3.5.2).

## Additional files

Additional file 1: **Table S1.** Barcodes. Forward and reverse barcode sets used for BART-Seq experiments (a) barcodes v1, (b) NNN barcodes, (c) barcodes v2 (XLSX 20 kb)

Additional file 2: **Table S2.** Primers and loci. Primers used for BART-Seq experiments and the list of reported mutations for 96 patient samples used in the study. (a) Genotyping primers, (b) reported genotypes of the patient samples, (c) primers used with NNN barcodes, (d) pluripotency primers, (e) differentiation primers (XLSX 45 kb)

Additional file 3: **Table S3.** Genotyping experiment. De-multiplexed NGS read counts of the *BRCA* genotyping NGS experiments. (a) Genotyping using barcodes without a protection group, (b) frequencies of NNN protection groups, (c) genotyping using barcodes with 5′CCA protection group (XLSX 74 kb)

Additional file 4: **Table S4.** RNA dilution experiment. De-multiplexed NGS read counts of the RNA/cDNA dilution experiments starting from (a) RNA or (b) cDNA templates (XLSX 32 kb)

Additional file 5: **Table S5.** Growth media experiment. De-multiplexed NGS read counts of the media comparison experiment. (a) Replicate 1 and (b) replicate 2 (XLSX 662 kb)

Additional file 6: **Table S6.** Wnt experiment. De-multiplexed NGS read counts of the differentiation (Wnt/β-catenin) experiment. (a) Replicate 1, (b) replicate 2, (c) replicate 3 (XLSX 851 kb)

Additional file 7: **Figure S1.** Supporting evidence regarding barcode assembly, gBART-Seq, and protection groups, related to main Figs. 1 and 2. **Figure S2.** Supporting evidence for RNA quantification experiments, related to main Fig. 3. **Figure S3.** Using spike-ins for read normalizing, related to main Figs. 3, 4, and 5. **Figure S4.** Supporting evidence of the growth media comparison experiment, related to Fig. 4. **Figure S5.** Supporting evidence of Wnt/β-catenin pathway manipulation, related to Fig. 5 (PDF 20474 kb)

## Data Availability

Data: The raw and processed BART-Seq data discussed in this manuscript is deposited in NCBI’s Gene Expression Omnibus (NCBI-GEO) and is accessible under SuperSeries: GSE107723 (https://www.ncbi.nlm.nih.gov/geo/query/acc.cgi?acc=GSE107723) [51]. Bulk RNA sequencing data used for comparison to 72 h samples (bCat: GSM3737181, GSM3737182; CHIR99021: GSM3737193, GSM3737194; rWnt3a: GSM3737203, GSM3737204) is available under: GSE130381 (https://www.ncbi.nlm.nih.gov/geo/query/acc.cgi?acc=GSE130381) [52]. Codes: The scripts for designing barcodes and primers and normalizing the read counts are available at https://github.com/theislab/bartSeq, licensed under GNU General Public License v3.0 [53]. The versions used in this manuscript are permanently available under 10.5281/zenodo.3252205. The pipeline for demultiplexing the sequencing reads are available at https://github.com/theislab/bartseq-pipeline, licensed under GNU General Public License v3.0 [54]. The version used in this manuscript is permanently available under 10.5281/zenodo.3251773. The website for designing the primers is available at http://icb-bar.helmholtz-muenchen.de.

## Abbreviations

cDNA: Complementary DNA
Ct: Cycle threshold
Dox: Doxycycline
FACS: Fluorescence-activated cell sorting
gDNA: Genomic DNA
GSK3: Glycogen synthase kinase 3
hESCs: Human embryonic stem cells
hPSCs: Human pluripotent stem cells
lncRNA: Long non-coding RNA
MEFs: Mouse embryonic fibroblasts
mfe: Minimum free energy
MIP: Molecular inversion probe
NGS: Next-generation sequencing
qPCR: Quantitative polymerase chain reaction
rc: Reverse complementary
RT: Reverse transcription
rWnt3a: Recombinant Wnt3a
tSNE: *t*-distributed stochastic neighbor embedding
UMAP: Uniform manifold approximation and projection
UMI: Unique molecular identifier
λ-exo: Lambda exonuclease
