# Supplementary material for: BART-Seq: cost-effective massively parallelized targeted sequencing for genomics, transcriptomics, and single-cell analysis
Source: Genome Biol. 2019 Aug 6;20:155. doi: 10.1186/s13059-019-1748-6 (PMC6683345; doi:10.1186/s13059-019-1748-6)
Supplement: Supplementary file 7 — Figure S1. Supporting evidence regarding barcode assembly, gBART-Seq, and protection groups, related to main Figs. 1 and 2. Figure S2. Supporting evidence for RNA quantification experiments, related to main Fig. 3. Figure S3. Using spike-ins for read normalizing, related to main Figs. 3, 4, and 5. Figure S4. Supporting evidence of the growth media comparison experiment, related to Fig. 4. Figure S5. Supporting evidence of Wnt/β-catenin pathway manipulation, related to Fig. 5 (PDF 20474 kb) [file 13059_2019_1748_MOESM7_ESM.pdf]

Figure S1

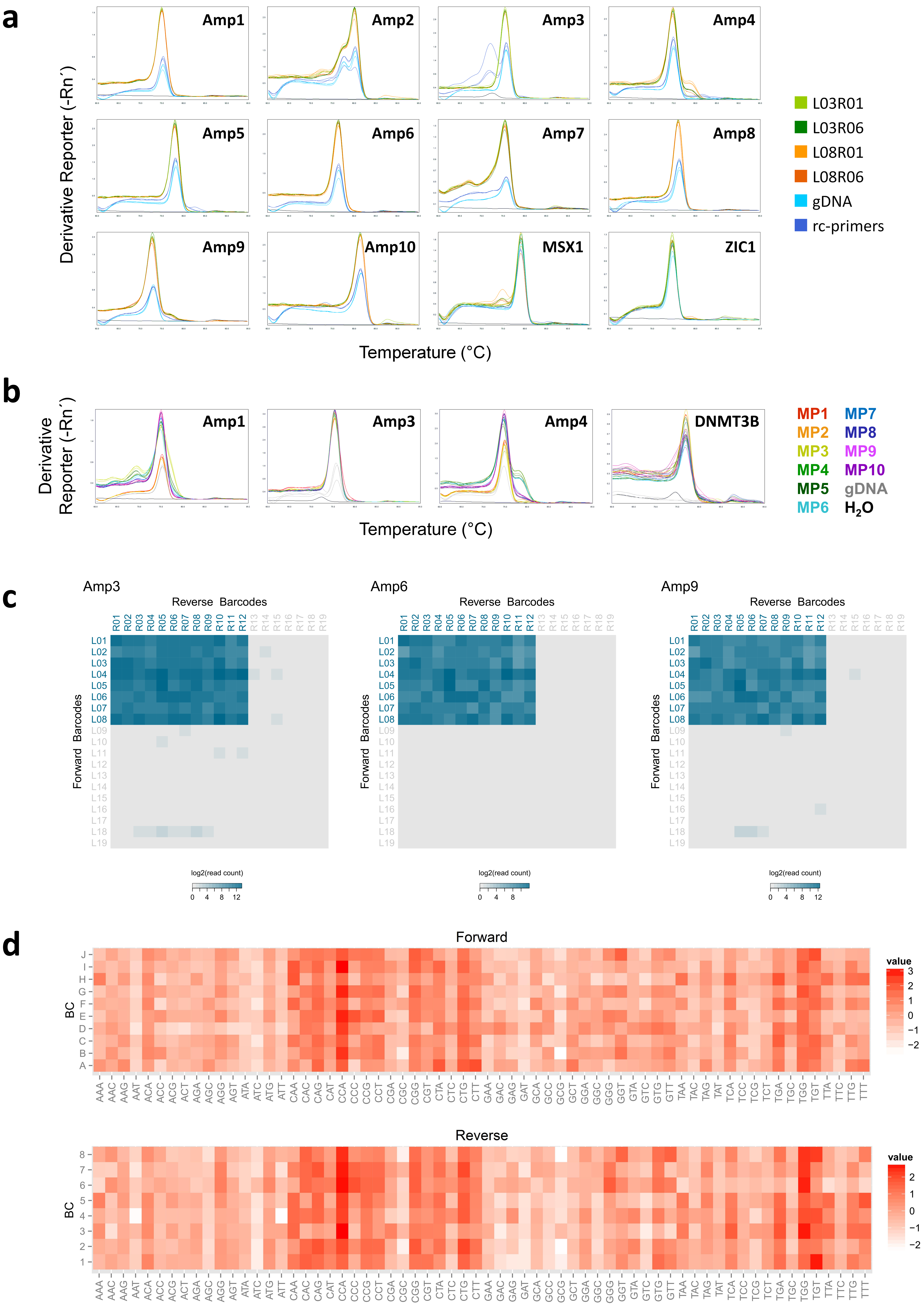
a
Derivative Reporter (-Rn´)
Amp1
Amp2
Amp3
Amp4
Amp5
Amp6
Amp7
Amp8
Amp9
Amp10
MSX1
ZIC1
Temperature (°C)
L03R01
L03R06
L08R01
L08R06
gDNA
rc-primers
b
Derivative Reporter (-Rn´)
Amp1
Amp3
Amp4
DNMT3B
Temperature (°C)
MP1
MP2
MP3
MP4
MP5
MP6
MP7
MP8
MP9
MP10
gDNA
H2O
c
Amp3
Amp6
Amp9
Reverse Barcodes
Forward Barcodes
log2(read count)
d
Forward
Reverse
BC
value

Figure S1 | **Supporting evidence regarding barcode assembly, gBART-Seq, and protection groups, related to main Figure 1 and Figure 2**. **(a)** Melting curve analysis of amplicons in **Fig. 1d** grouped according to the loci (Amp1-10). Non-pre-amplified samples (gDNA, light blue), non-barcoded rc-primers (dark blue), and non-targeted loci (*MSX1* and *ZIC1*) exhibited weaker signals and higher Ct values compared to the samples pre-amplified with barcoded primers. **(b)** Melting curves of the amplicons shown in **Fig. 1e**. The melting curve signals of Amp3 was the same for the range of multiplexing 1 to 10 primer pairs, while Amp1 and Amp4 exhibited weak signals in samples where their primers were absent in the pre-amplification step (MP1-2 and MP1-3, respectively). The non-targeted *DNMT3B* locus, non-pre-amplified gDNA (gray), and water (H_2O, black line) were negative controls. **(c)** Heatmaps showing the reads assigned to three out of ten *BRCA* amplicons (Amp3, Amp6, and Amp9) (sum of the wild-type and mutation reads) in the matrix of the analyzed samples as in **Fig. 2b**. **(d)** Evaluation of protection effects of trinucleotides. A matrix of ten forward (A-J) and eight reverse (1-8) barcodes were used with flaking 5'NNN trinucleotides for barcoding the *BRCA* genotyping primer set (**Fig. 2, Additional file 2: Table S2a**) with constant amount of gDNA template from MCF7 cells. Heatmaps illustrating the sorted amplicons to each forward and reverse barcode, and relative frequency of 5' trinucleotide sequences indicate that 5'CCA is one of the most abundant trinucleotides, meaning most resistant to λ-exo digestion. Intensity range light to dark corresponds to low to high read numbers respectively.

Figure S2

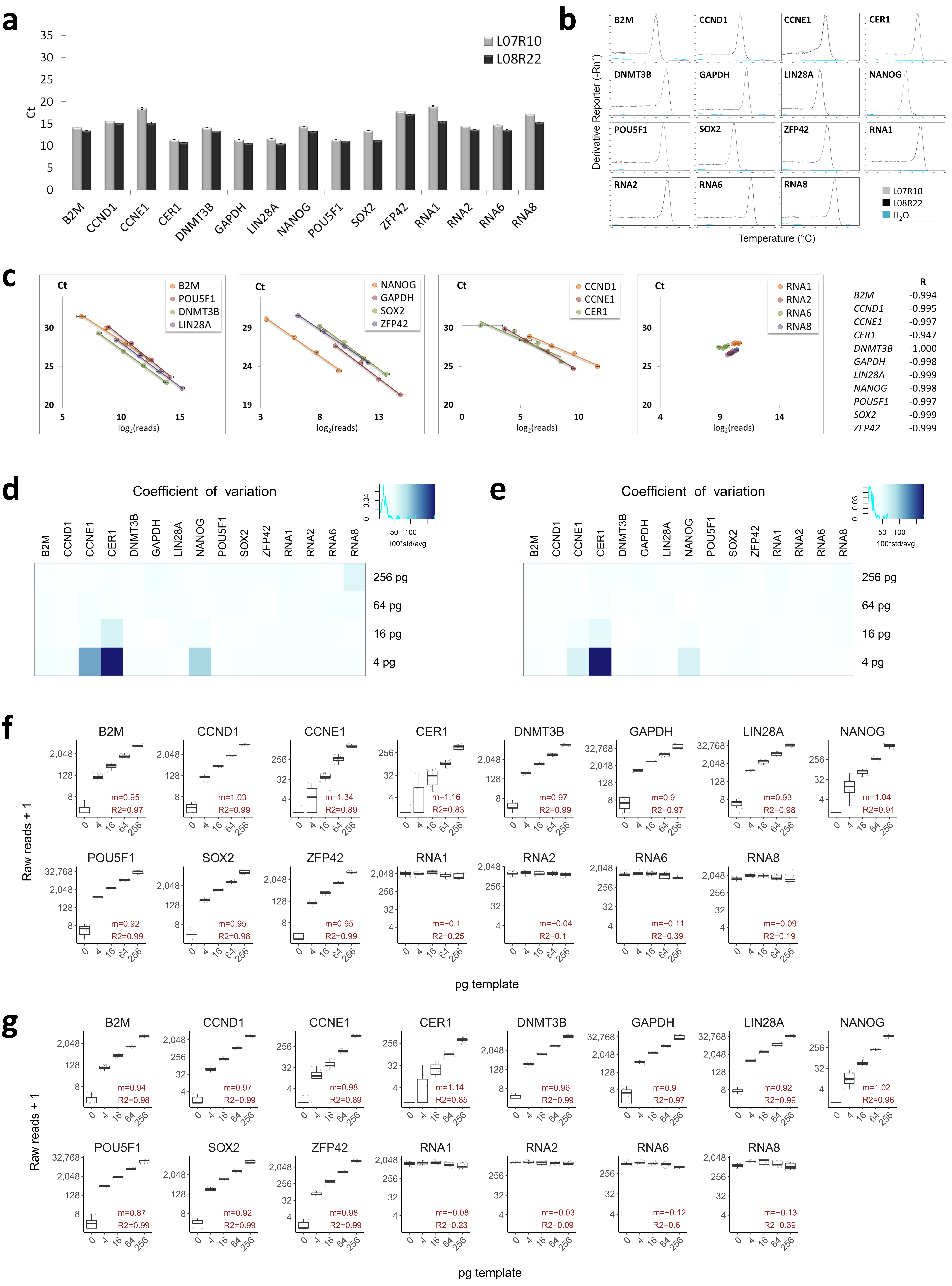

a
L07R10
L08R22
Ct
B2M
CCND1
CCNE1
CER1
DNMT3B
GAPDH
LIN28A
NANOG
POU5F1
SOX2
ZFP42
RNA1
RNA2
RNA6
RNA8
b
Derivative Reporter (-Rn')
Temperature (°C)
L07R10
L08R22
H2O
c
Ct
log2(reads)
R
B2M -0.994
CCND1 -0.995
CCNE1 -0.997
CER1 -0.947
DNMT3B -1.000
GAPDH -0.998
LIN28A -0.999
NANOG -0.998
POU5F1 -0.997
SOX2 -0.999
ZFP42 -0.999
d
Coefficient of variation
100*std/avg
256 pg
64 pg
16 pg
4 pg
e
Coefficient of variation
100*std/avg
256 pg
64 pg
16 pg
4 pg
f
Raw reads + 1
pg template
B2M m=0.95 R2=0.97
CCND1 m=1.03 R2=0.99
CCNE1 m=1.34 R2=0.89
CER1 m=1.16 R2=0.83
DNMT3B m=0.97 R2=0.99
GAPDH m=0.9 R2=0.97
LIN28A m=0.93 R2=0.98
NANOG m=1.04 R2=0.91
POU5F1 m=0.92 R2=0.99
SOX2 m=0.95 R2=0.98
ZFP42 m=0.95 R2=0.99
RNA1 m=−0.1 R2=0.25
RNA2 m=−0.04 R2=0.1
RNA6 m=−0.11 R2=0.39
RNA8 m=−0.09 R2=0.19
g
Raw reads + 1
pg template
B2M m=0.94 R2=0.98
CCND1 m=0.97 R2=0.99
CCNE1 m=0.98 R2=0.89
CER1 m=1.14 R2=0.85
DNMT3B m=0.96 R2=0.99
GAPDH m=0.9 R2=0.97
LIN28A m=0.92 R2=0.99
NANOG m=1.02 R2=0.96
POU5F1 m=0.87 R2=0.99
SOX2 m=0.92 R2=0.99
ZFP42 m=0.98 R2=0.99
RNA1 m=−0.08 R2=0.23
RNA2 m=−0.03 R2=0.09
RNA6 m=−0.12 R2=0.6
RNA8 m=−0.13 R2=0.39

Figure S2 | **Supporting evidence for RNA quantification experiments, related to main Figure 3. (a-c)** Nested qPCR-based assessment of the pluripotency primer set. **(a)** Primer sets targeting 11 genes and 4 RNA spike-ins were assembled with two different barcode combinations (L07×R10 and L08×R22) and used for pre-amplification of hESC cDNA template (100 pg/µl per reaction). Shown are the Ct values of pre-amplified samples assessed by nested primers (**Additional file 2: Table S2d**) in duplicates, and error bars represent standard deviations. **(b)** Melting curve analysis of the qPCR in **a**. **(c)** cDNA dilution series that was analyzed by rBART-Seq as described in **Fig. 3a** was in parallel analyzed by qPCR. Presented are the correlation plots between average log2 read counts per concentration versus the Ct values per gene. Error bars are the standard deviation of replicate samples, vertical representing three qPCR replicates, and horizontal representing nine sequencing replicates harboring different barcode combinations. R values on the right are the corresponding correlation coefficients. **(d-e)** Heatmaps showing coefficient of variations calculated using raw reads of libraries produced using (**d**) RNA template (related to **Fig. 3b**) or (**e**) cDNA template (where bulk RNA and spike-in mixtures were converted to cDNA before aliquoting), based on groups of nine replicates harboring different barcode combinations (**Additional file 4: Table S4**). RNA8 in **d** had one outlier well in 256 pg samples. **(f-g)** Boxplots showing the raw read counts assigned to 11 transcripts and 3 RNA spike-ins, plotted against the template concentration, for (**f**) RNA and (**g**) cDNA libraries. Slopes (m) were close to 1 for the majority of samples, and coefficients of determination (R^2) were higher than 0.96 on average, in the linear regression models calculated for the 4-256 pg sample groups using log2 read counts.

Figure S3

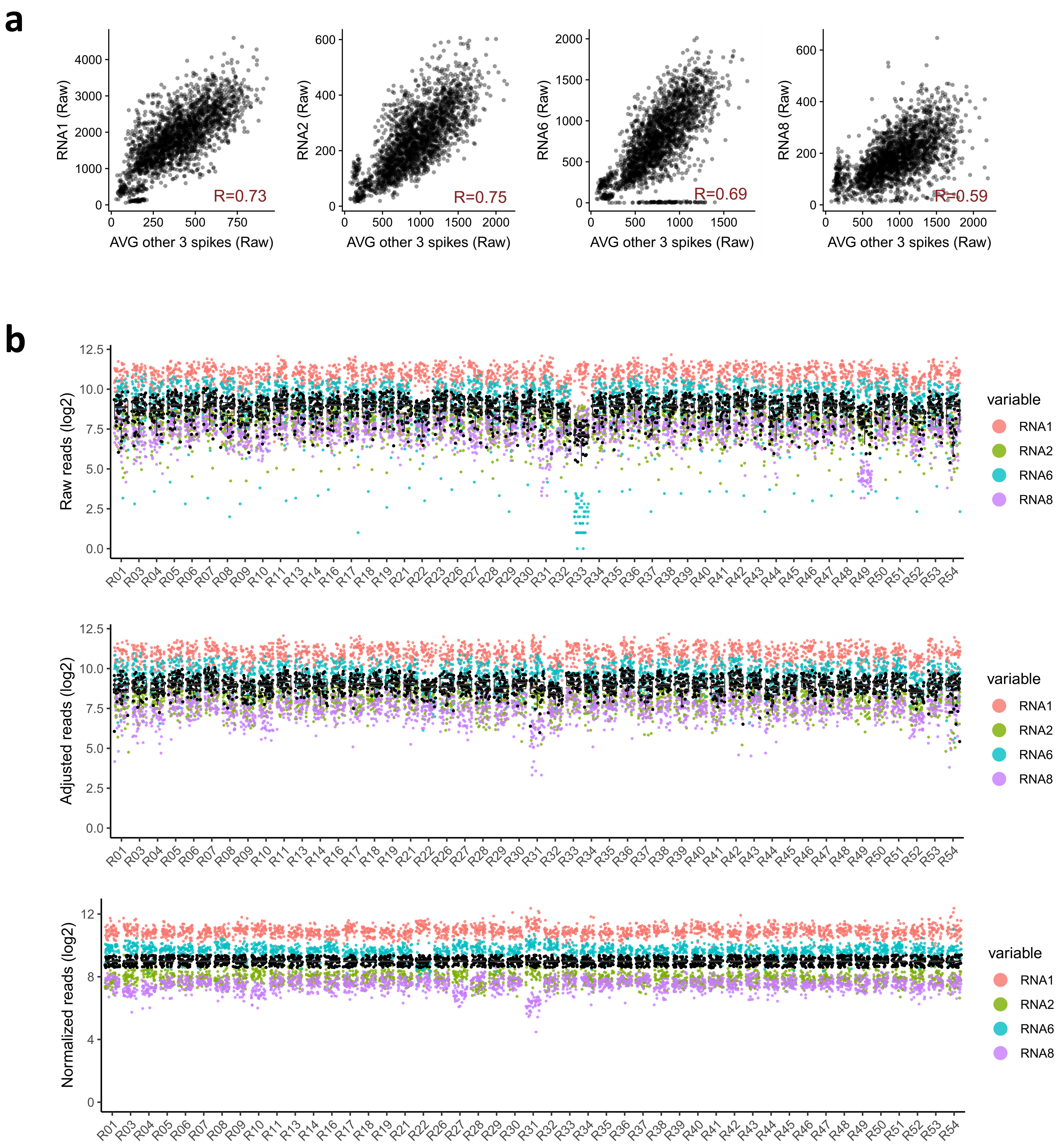
a
RNA1 (Raw)
AVG other 3 spikes (Raw)
R=0.73
RNA2 (Raw)
R=0.75
RNA6 (Raw)
R=0.69
RNA8 (Raw)
R=0.59
b
Raw reads (log2)
Adjusted reads (log2)
Normalized reads (log2)
variable
RNA1
RNA2
RNA6
RNA8

Figure S3 | Using spike-ins for read normalizing, related to main Figures 3-5. Four spike-in RNAs were included in transcriptomics experiments. They were added to templates before RT step for controlling technical variations. **(a)** Raw reads (from an experiment with 2304 single cell samples, **Additional file 5: Table S5a**) assigned to each spike-in correlated with the average of the other three spike-ins, suggesting that their variation mainly reflect technical variations rather than stochastic effects. Correlation coefficients (R) are shown in the bottom. **(b)** An example of the normalization process (from **Additional file 5: Table S5a**). Specific barcode-primer combinations cause extremely low reads occasionally, which might bias the scaling factors calculated using the spike-ins, as shown with shifted black dots relative to the average in the upper panel (e.g. barcodes R33 and R49), and in turn might cause over-correction of these samples. Before data normalization, such combinations were identified with t-test and replaced with the median of the rest of the barcodes to avoid this bias (middle panel). Following normalization of the whole data for technical variations using a script that calculates scaling factors based on spike-in reads (and genes, in some cases), variation among spike-in reads decreased to a 2-fold range (lower panel) (details in **Online Methods**)

Figure S4

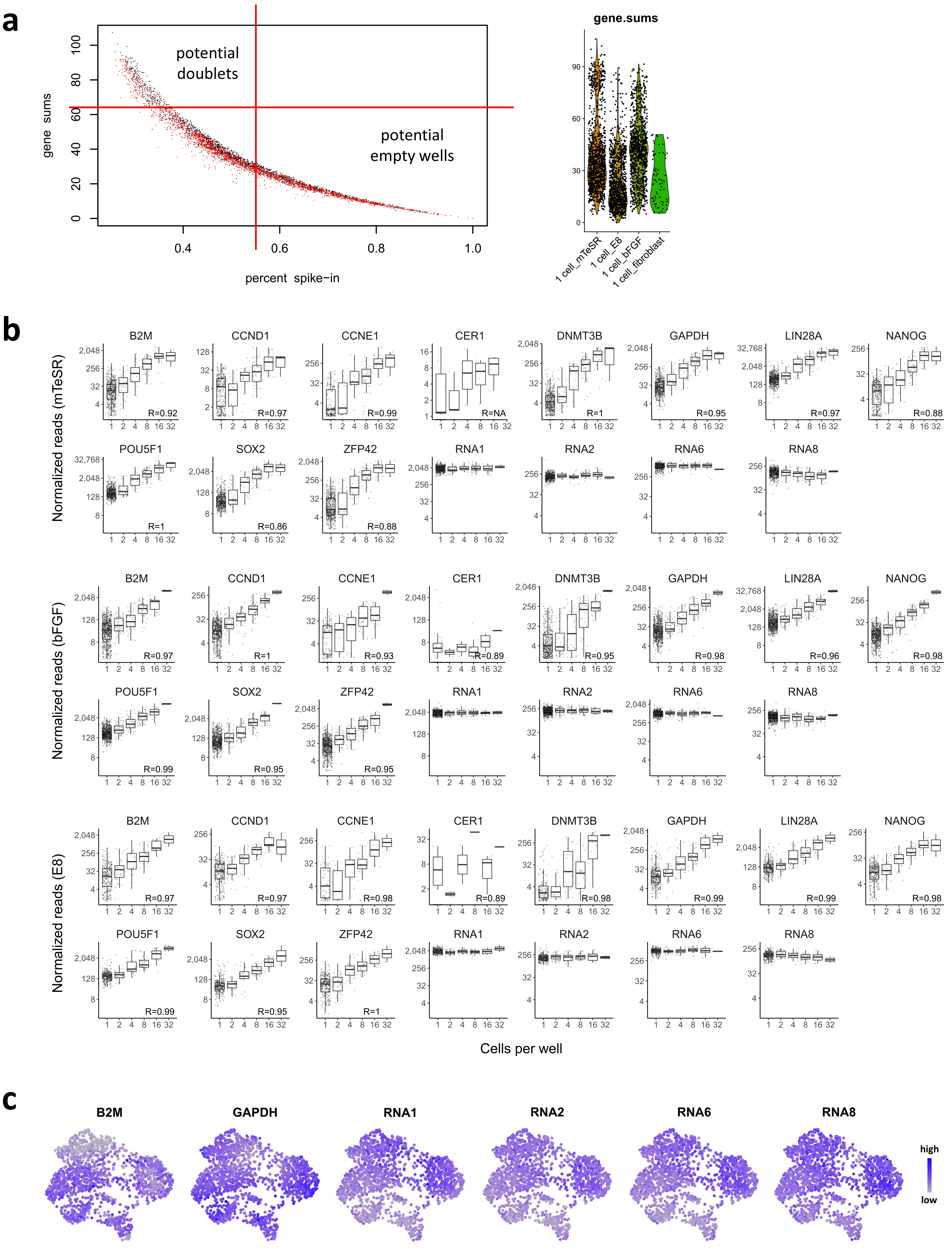
a
potential doublets
potential empty wells
gene sums
percent spike−in
gene.sums
1 cell_mTeSR
1 cell_E8
1 cell_bFGF
1 cell_fibroblast
b
Normalized reads (mTeSR)
B2M R=0.92
CCND1 R=0.97
CCNE1 R=0.99
CER1 R=NA
DNMT3B R=1
GAPDH R=0.95
LIN28A R=0.97
NANOG R=0.88
POU5F1 R=1
SOX2 R=0.86
ZFP42 R=0.88
RNA1
RNA2
RNA6
RNA8
Normalized reads (bFGF)
B2M R=0.97
CCND1 R=1
CCNE1 R=0.93
CER1 R=0.89
DNMT3B R=0.95
GAPDH R=0.98
LIN28A R=0.96
NANOG R=0.98
POU5F1 R=0.99
SOX2 R=0.95
ZFP42 R=0.95
RNA1
RNA2
RNA6
RNA8
Normalized reads (E8)
B2M R=0.97
CCND1 R=0.97
CCNE1 R=0.98
CER1 R=0.89
DNMT3B R=0.98
GAPDH R=0.99
LIN28A R=0.99
NANOG R=0.98
POU5F1 R=0.99
SOX2 R=0.95
ZFP42 R=1
RNA1
RNA2
RNA6
RNA8
Cells per well
c
B2M
GAPDH
RNA1
RNA2
RNA6
RNA8
high
low

Figure S4 | **Supporting evidence of the growth media comparison experiment, related to Figure 4. (a)** Sample filtering was based on the ratio of spike-in reads to the total reads per sample, as well as sum of the gene reads (log2 values). Thresholds were calculated based on negative control wells without cells. Samples with spike-in percentage higher than the threshold were treated as “empty” and removed. Samples with exceptionally high total gene sums (potential doublets) were removed based on threshold determined by the bimodal distribution (right panel). **(b)** Normalized read counts for the entire panel of pluripotency gene panel plotted per condition with respect to the number of cells sorted to each well (n=675, n=858, and n=427 samples for mTeSR™1, KSR-bFGF, and E8 respectively) (related to **Fig. 4b**). Correlation coefficients (R) between the cell counts and the median of corresponding reads are shown. **(c)** Expression of the rest of the genes underlying the UMAP projection in **Fig. 4d**.

Figure S5

a

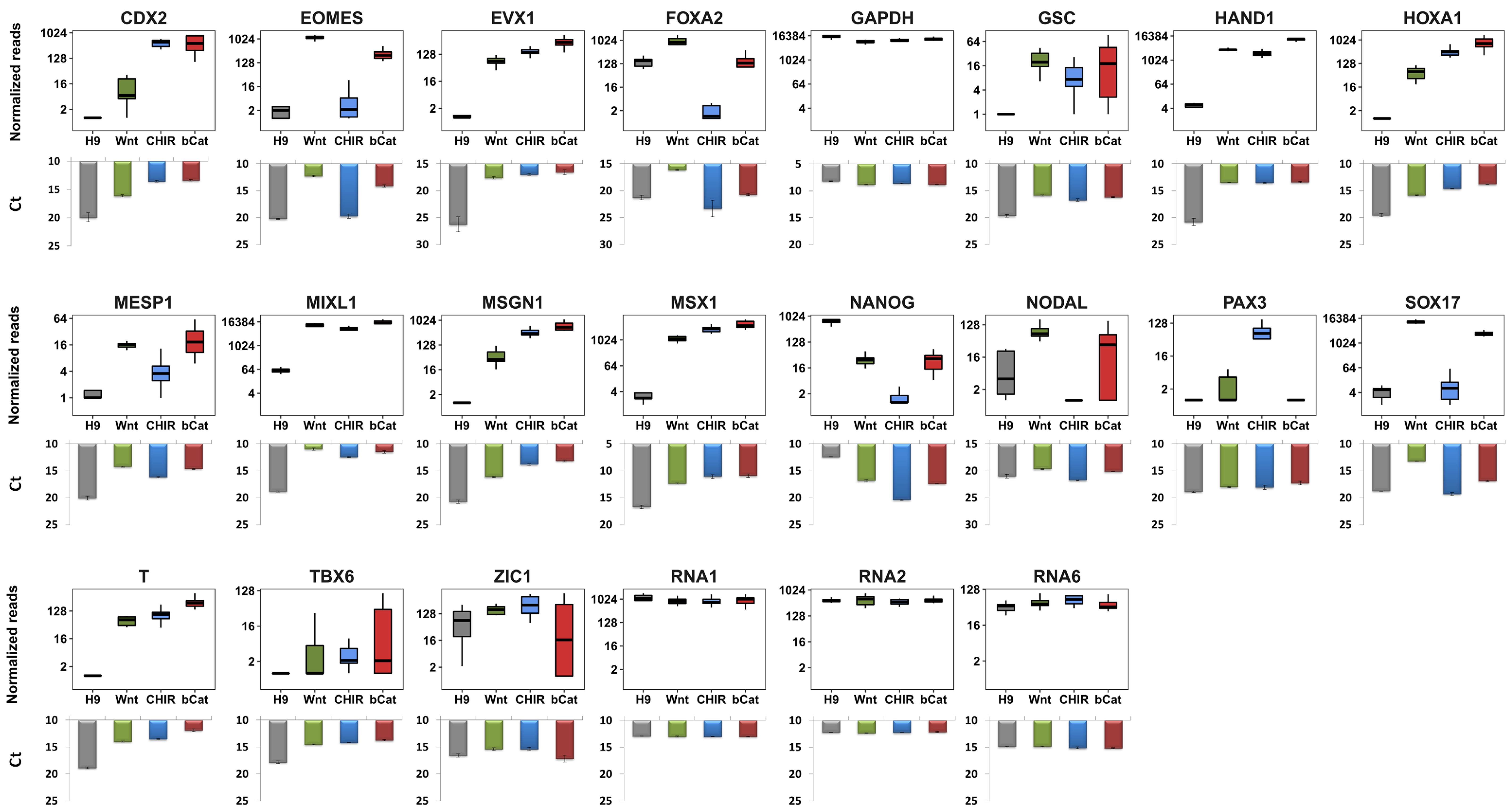

b

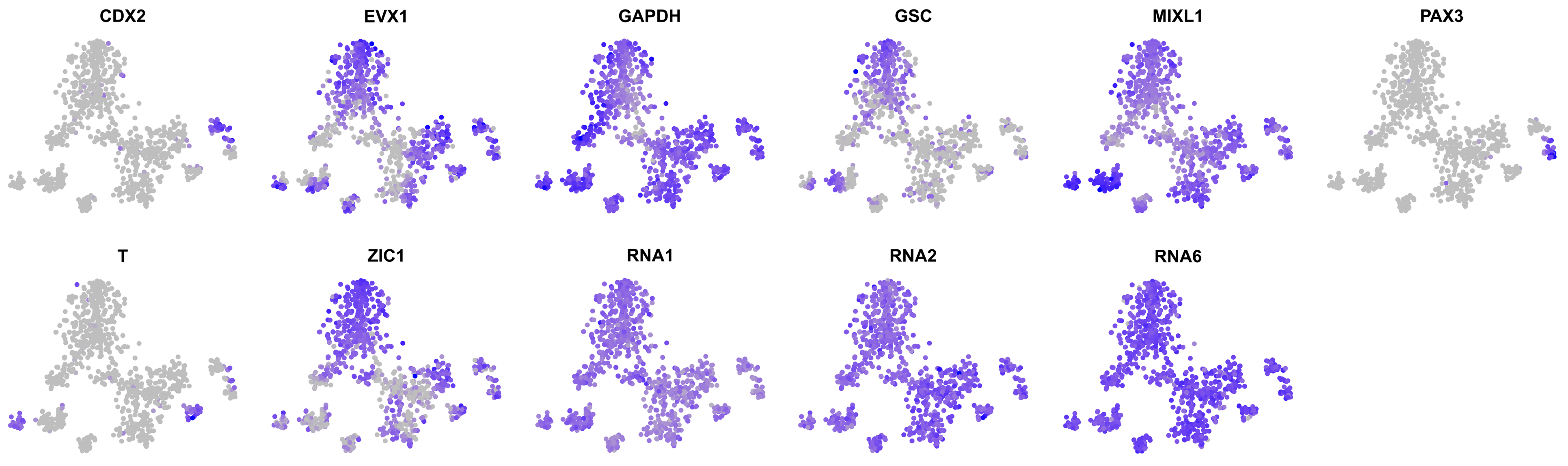

c

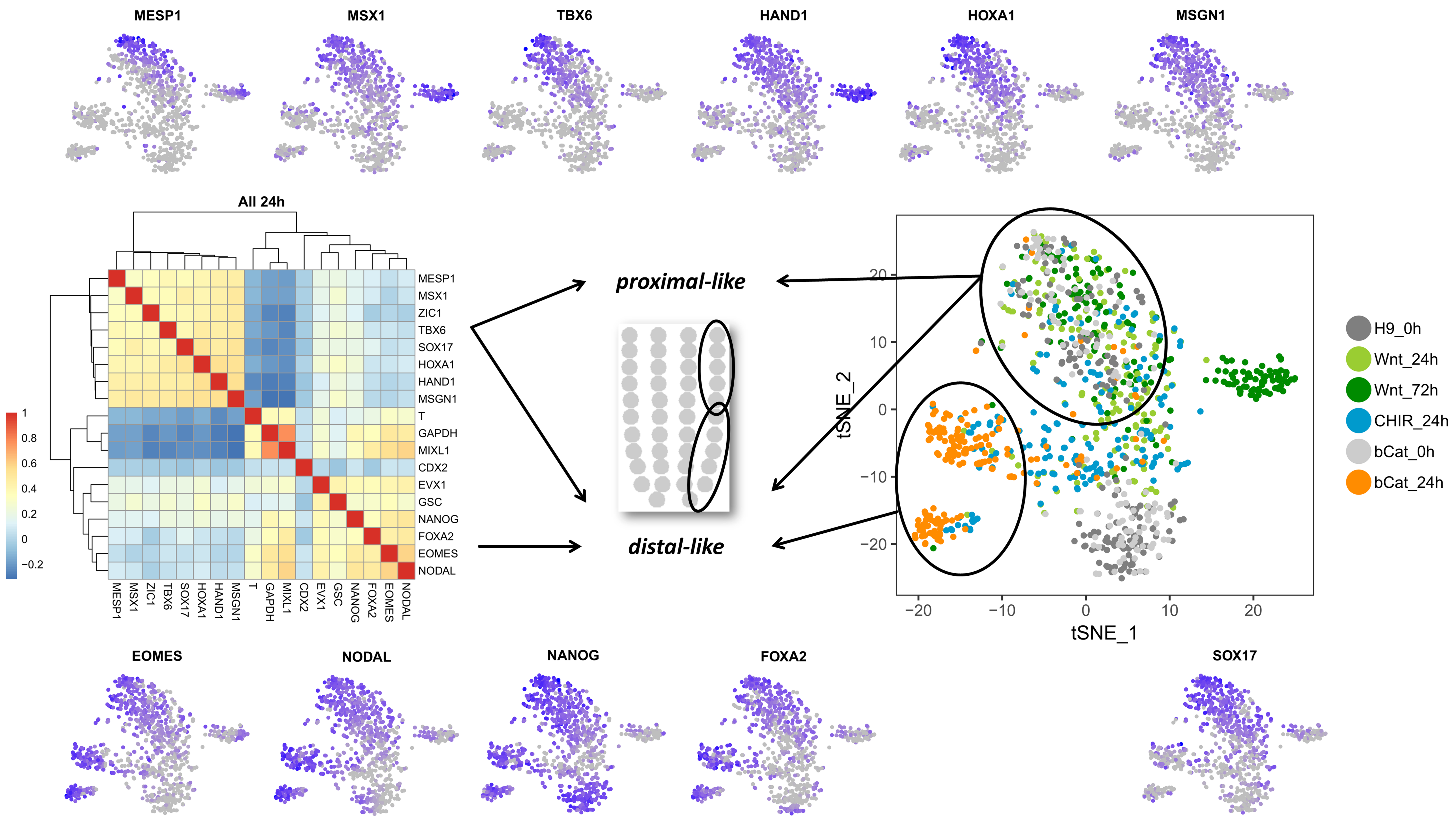

Figure S5 | **Supporting evidence of Wnt/β-catenin pathway manipulation, related to Figure 5.** **(a)** rBART-Seq and qPCR analysis of bulk RNA samples isolated from undifferentiated hESCs and after 72 hours of treatment by three factors as in **Fig. 5.** Upper panels show the normalized reads from RNA samples that were aliquoted (50 pg per well), barcoded with 10 different combinations, and analyzed together with single cells. Lower panels show the Ct values from nested primer assessment of bulk samples from a biological repetition after multiplex PCR using differentiation primers, displaying striking similarity with the rBART-Seq results. **(b)** Expression of the rest of the genes underlying the tSNE distribution in **Fig. 5**. **(c)** A biological repetition of the experiment in **Fig. 5**. Heatmap of the pairwise gene correlations calculated based on single cells after 24 hours from the three treatments (left), and two dimensional representation (tSNE) of the single cells sampled at 0, 24, and 72 hours from all treatments, based on the expression of 19 genes (right). The 72h timepoint contains cells only from Wnt3a treatment due to the loss of samples from the other two conditions. Distribution of some selected genes underlying the tSNE plot is shown in the upper and lower panels. Relevant corn plots demonstrating the expression of selected genes in the epiblast of gastrulating mouse embryo (E6.5-E7.5) based on the iTranscriptome database (34) is presented in the main **Fig. 5.**
